# The Role of Interferon-Gamma in Hematopoietic Stem Cell Development, Homeostasis, and Disease

**DOI:** 10.1007/s40778-018-0139-3

**Published:** 2018-07-23

**Authors:** Daniel E. Morales-Mantilla, Katherine Y. King

**Affiliations:** 1 Program in Immunology and Center for Cell and Gene Therapy, Section of Infectious Diseases, Department of Pediatrics, Baylor College of Medicine, Houston, TX 77030 USA; 2 Pediatric Infectious Diseases, Baylor College of Medicine, 1102 Bates St. Suite 1150, Houston, TX 77030 USA

**Keywords:** Interferon-gamma, Hematopoietic stem cells, Bone marrow, Terminal differentiation, Emergency hematopoiesis, Bone marrow failure

## Abstract

**Purpose of Review:**

Interferon-gamma (IFN-γ) is a pro-inflammatory cytokine that participates in the regulation of hematopoietic stem cells (HSC) during development and under homeostatic conditions. IFN-γ also plays a key pathogenic role in several diseases that affect hematopoiesis including aplastic anemia, hemophagocytic lymphohistiocytosis, and cirrhosis of the liver.

**Recent Findings:**

Studies have shown that increased IFN-γ negatively affects HSC homeostasis, skewing HSC towards differentiation over self-renewal and eventually causing exhaustion of the HSC compartment.

**Summary:**

Here, we explore the mechanisms by which IFN-γ regulates HSC in both normal and pathological conditions. We focus on the role of IFN-γ signaling in HSC fate decisions, and the transcriptional changes it elicits. Elucidating the mechanisms through which IFN-γ regulates HSCs may lead to new therapeutic options to prevent or treat adverse hematologic effects of the many diseases to which IFN-γ contributes.

## Introduction

**Fig. 1:**
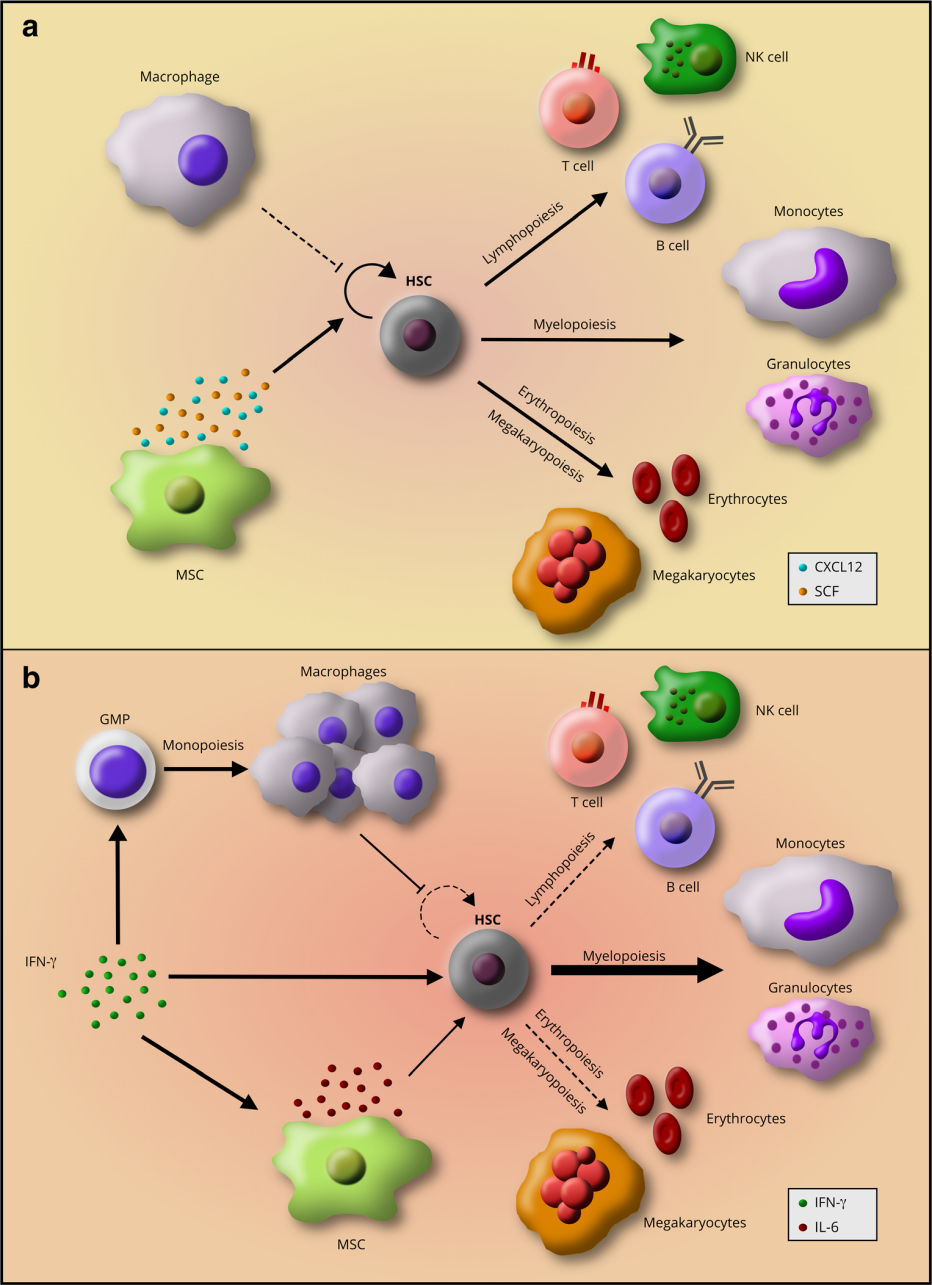
The self-renewal and differentiation equilibrium of HSCs in their niche. **a** Steady-state hematopoiesis is a balance between HSC self-renewal and differentiation. Cues from the niche regulate this balance to maintain HSC quiescence and self-renewal capabilities while contributing to the production of differentiated immune and blood cells. **b** IFN-γ signaling alters hematopoiesis and niche cues by inducing myeloid-biased HSC differentiation at the expense of self-renewal and differentiation to the lymphoid and erythroid lineages. IFN-γ also suppresses HSC self-renewal either directly or by contributing to changes in niche cells such as production of more macrophages and induction of IL-6 production by MSCs

Homeostatic production of blood and immune cells by hematopoietic stem and progenitor cells (HSPC) in the bone marrow (BM) is necessary to support tissue oxygenation and immunity (Fig. 1a). Recent developments in hematopoietic stem cell (HSC) research have shown that inflammatory cytokines can affect blood production by the bone marrow. HSCs express cytokine receptors and pattern-recognition receptors (PRR) and can directly respond to pro-inflammatory cytokines and pathogen-associated molecular patterns (PAMPs) [1–4]. These observations have led to a new understanding of the role of both tonic and stress inflammatory signals in regulation and homeostasis of the HSC pool.

Interferon-gamma (IFN-γ) is a type II interferon secreted by T cells and NK cells during Th1-mediated immune responses. IFN-γ functions as a pro-inflammatory cytokine that mediates antimicrobial, antiviral, and antitumor responses by activating effector immune cells and enhancing antigen presentation [5]. This cytokine is known to play a role in a number of diseases in which HSPCs are impaired including aplastic anemia, hemophagocytic lymphohistiocytosis (HLH), cirrhosis of the liver, and many infectious diseases such as HIV, mycobacterial infections, and hepatitis C [4, 6–15]. Recent studies have shown that IFN-γ can affect HSCs at multiple levels, ranging from their development, quiescence, and differentiation, to the niche cells that support them [16•, 17•, 18••, 19•, 20–22, 23•, 24••, 25, 26••]. Increasing our understanding of how IFN-γ regulates HSCs may identify pathways to improve homeostasis of the hematopoietic system and immunity during inflammatory stress.

In this review, we summarize the effects of IFN-γ on HSC development, homeostasis, and fate. We focus on the influence of IFN-γ signaling on HSC differentiation and how it can lead to irreversible HSC depletion.

## IFN-γ Signaling in HSC Development

In vertebrates, HSCs arise from endothelial cells (EC) in hemogenic areas such as the aorta-gonad-mesonephros (AGM) region of the embryo [17•, 27]. The signaling pathways promoting HSC emergence are largely shared across different vertebrates [17•, 28]. Studies have shown that the mechanical forces from blood flow are necessary for HSC emergence [17•, 27, 29, 30]. Blood flow induces the expression of IFN-γ, IFN-γ receptor (IFNγR), and Runx1 [17•, 29], an essential regulator of the EC-to-HSC transition [31] and HSC emergence [27]. ECs in the AGM respond to IFN-γ, leading to downstream signaling through the signal transducer and activator of transcription 3 (STAT3) [17•] to promote the expression of genes responsible for the EC-to-HSC transition. Several groups have demonstrated that sterile tonic inflammatory signaling is required for HSC emergence [16•, 17•, 32–35, 36•]. Thus, IFN-γ signaling through non-canonical STAT3 activation is required for normal HSC development at the embryonic stage.

## IFN-γ and the Cell Cycle

The importance of IFN-γ signaling in HSCs is not limited to their development. Recent evidence has shown that IFN-γ regulates HSC cell cycle activity. Baldridge et al. reported that HSCs are stimulated to proliferate during *Mycobacterium avium* infection via an IFN-γ-dependent mechanism, and a similar phenomenon has also been demonstrated during *Ehrlichia muris* infection [20, 22]. Type I interferons (IFN-α and IFN-β) similarly induce HSCs to exit from dormancy [20, 37]. In contrast, another study indicated that IFN-γ reduces HSC proliferation in vivo and that in vitro culturing of HSCs with IFN-γ did not induce HSCs to come out of quiescence [38]. While some have suggested that these conflicting findings might be explained by Sca-1 induction by interferon in myeloid progenitor cells (which normally do not express this marker and can thus be falsely reported as HSCs [18••, 22, 23•, 39]), the induction of HSC proliferation in response to IFN-γ has since been documented using a range of different surface markers and avoiding Sca-1 as an HSC marker. Differences in experimental conditions are more likely to explain the different conclusions of these studies, as we discuss below. Furthermore, inflammation-dependent activation of HSC proliferation has been confirmed using methods that do not depend on cell surface marker expression [1, 37]. Altogether, the accumulated evidence indicates that IFN-γ induces HSCs to exit their quiescent state.

## IFN-γ and Cell Fate

### Apoptosis

While the short-term effects of IFN-γ signaling are characterized by an increase in HSC proliferation, the long-term effects appear to be deleterious, leading to HSC depletion and exhaustion. Interferons are well known to trigger apoptosis in many cell types including cancer cells [40–42] and whether IFN-γ directly induces HSC apoptosis has been intensely debated. In vitro IFN-γ stimulation of human CD34+ HSCs co-cultured with stromal cells increased HSC apoptosis [43]. Furthermore, transcriptional profiling of HSCs from patients with high IFN-γ levels have indicated transcriptional signatures of apoptosis [44]. However, more recently, cultured primitive HSCs stimulated with IFN-γ showed no increase in apoptosis [20]. Additionally, mice infected with *M. avium* or directly treated with IFN-γ did not show increased HSC apoptosis, although these cells were more sensitive to undergo apoptosis after a secondary stress [18••]. This sensitization to apoptosis has also been reported upon stimulation with other pro-inflammatory cytokines, such as IFN-α [39]. Indeed, HSC exit from the quiescent state in response to either IFN-α or IFN-γ is accompanied by increased DNA damage [1, 37, 45], thus providing a possible explanation by which the threshold for apoptosis is lowered. Strikingly, HSC apoptosis increases in pro-inflammatory diseases concurring with high levels of IFN-γ in the circulation, such as aplastic anemia (AA) [4, 44]. However, IFN-γ is not the only cytokine that is upregulated in these diseases. IFN-γ signaling in AA stimulates T cells to produce TNF-α and RANKL [46], two molecules known to promote apoptosis. Similarly, in patients with liver cirrhosis, blood levels of IFN-γ, TNF-α, IL-1β, and IL-6 also increase, whereas the total number of CD34+ HSCs decreases in proportion to disease severity [8]. Interestingly, in a mouse model of lymphochoriomeningitis virus (LCMV) infection, IFN-γ signaling resulted in overall reduced BM cellularity [19•, 38]. LCMV infection induces the production of multiple cytokines, including IFN-α, IL-1, and IL-6, in addition to IFN-γ, and has been reported to induce NLRP1 inflammasome-mediated pyroptosis of hematopoietic progenitor cells [47, 48]. Altogether, these studies suggest that IFN-γ only triggers HSC apoptosis in the presence of other stress stimuli.

Consistent with this theme, Chen et al. showed that IFN-γ induces the expression of Fas and pro-apoptotic caspases in HSPC; however, in this study, the HSPCs only underwent apoptosis when cultured with activated cytotoxic T cells [26••]. Fas upregulation has similarly been described in human CD34^+^ HSCs from AA patients [44] who also have autoreactive T cells that express FasL, contributing to the pathogenesis of AA and risk of bone marrow failure. Given the apparent requirement of additional cytokines for HSC apoptosis, the conflicting data between in vitro studies showing induction of apoptosis and in vivo studies showing no apoptosis is likely explained by the fact that taking HSCs out of their in vivo niche can be a primary stress by itself [18••]. Thus, by removing the cues that promote HSC maintenance, in vitro studies of cytokine stimulation inherently involve multiple stressors, and stimulation with IFN-γ, in addition to the stress of being outside the niche, can push HSCs into apoptosis. These observations indicate that IFN-γ does not directly induce apoptosis in HSCs, but rather that a combination of cytokines and stressors along with IFN-γ signaling can activate a pro-apoptotic response.

### Differentiation Versus Self-Renewal

In steady-state hematopoiesis, dividing HSCs either self-renew or differentiate. This balance allows for the maintenance of HSC numbers concomitantly with the production of necessary hematopoietic cells. However, IFN-γ signaling can alter this balance and push HSCs towards differentiation at the expense of self-renewal. Chen et al. showed that IFN-γ signaling downregulates *Gata2* and *Ets-1* expression in HSCs [26••]. These genes enforce HSC homeostasis, whereas decreased expression promotes differentiation [49, 50]. At the same time, IFN-γ signaling through STAT1 activates the expression of interferon regulatory factors (IRF) that can promote differentiation towards the myeloid lineage. For example, IRF1 activates PU.1, a master regulator of myeloid-promoting genes [51–53]. IFN-γ may also alter HSC differentiation by differential recruitment of lineage-biased HSC subtypes. Studies using LCMV showed that an HSC subtype biased towards the myeloid lineage (My-HSC) is preferentially recruited to differentiate at an accelerated rate in response to IFN-γ through the activation of *Cebpb* [19•], a transcription factor that promotes myeloid differentiation [54]. Infection-induced IFN-γ signaling promotes myelopoiesis in animal models of *Ehrlichia chaffeensis* and *M. avium* infection [19•, 21] as well as in vaccination with Bacillus Calmette-Guérin (BCG) [55••]. Furthermore, BCG vaccination reprograms HSCs to give rise to epigenetically altered macrophages with increased efficacy against TB infection [55••]. We previously conducted RNA sequencing analysis on purified HSCs from *M. avium*-infected mice to identify the drivers of differentiation during chronic infection [18••]. This study identified *Batf2*, also known as suppressor of AP-1, regulated by interferon (SARI), as an important mediator of myeloid differentiation. Deletion of *Batf2* interfered with myeloid differentiation both in mice infected with *M. avium* and human CD34+ HSCs stimulated with IFN-γ [18••]. Overall, these studies indicate that IFN-γ can drive gene expression changes in HSCs that promote their differentiation towards the myeloid lineage (Fig. 1b).

IFN-γ signaling also interferes with HSC self-renewal by upregulating the expression of the suppressor of cytokine signaling 1 (SOCS1) [56], which in turn inhibits STAT5 phosphorylation [38]. Thrombopoietin (TPO), a hematopoietic growth factor, is required to maintain HSC self-renewal properties through the activation of STAT5 after engaging its receptor c-MPL [57, 58]. Thus, by suppressing Stat5, IFN-γ impairs TPO signaling and thereby diminishes HSC self-renewal and reconstituting capabilities. Collectively, these observations show that IFN-γ promotes terminal differentiation directed by myeloid-promoting genes while simultaneously suppressing self-renewal pathways. These signaling changes provide insight into the potential mechanisms by which chronic IFN-γ stimulation depletes the HSC pool (Fig. 1b).

### Autophagy and Regulation of IFN-γ Responses

Autophagy has been described as an essential mechanism by which HSCs can survive nutrient deprivation and metabolic stress [59]. Interestingly, short-term IFN-γ signaling has been shown to induce expression of the autophagy-related p47 GTPase Irgm1 in HSCs. Irgm1-deficient mice demonstrate impaired autophagy flux in HSCs and a hyperproliferative phenotype associated with an expression signature of excessive IFN signaling [60]. These data suggest that autophagy can have a variety of context-specific roles in HSCs and may promote HSC longevity through regulation of IFN-γ signaling.

## IFN-γ and the HSC Niche

HSC homeostasis and maintenance are dependent on the tightly regulated microenvironment in which they reside. This microenvironment, or niche, is composed of multiple cell types including osteoblasts, endothelial cells, mesenchymal stromal cells (MSC), nerve fibers, and hematopoietic cells such as megakaryocytes and monocytes [61–64] which collectively provide an environment suitable for HSC homeostasis and survival. IFN-γ signaling alters these interactions. For example, HSPCs interact with their surrounding cells through expression of cell surface integrins. IFN-γ affects signaling by αvβ3 (CD51/CD61), an integrin that, in conjunction with TPO, promotes HSC maintenance, quiescence, and long-term repopulating activity in steady-state conditions [65, 66]. In the presence of IFN-γ stimulation, integrin αvβ3 signaling suppresses HSCs and causes a loss of long-term repopulating activity [25, 67]. These findings demonstrate that IFN-γ can change the outcome of integrin αvβ3 signaling from HSC maintenance to suppression.

IFN-γ affects several cells in the niche that maintain HSC homeostasis. Osteoblasts in the niche promote HSC maintenance through Notch signaling as well as through FasL-induced osteoclast apoptosis [62, 68, 69]. IFN-γ increases T cell production of TNFα and RANKL, which suppress osteoblast FasL expression, inducing bone resorption through increased osteoclast function [46, 68, 70]. In this process, T cell-produced TNF-α serves as a positive feedback loop that further downregulates osteoblastic FasL [68, 70], thus perpetuating a decline in osteogenesis and damage to the HSC niche. Similarly, type I interferons have been reported to damage the function of vascular endothelial cells, a critical component of the HSC niche [71]. Whether type II interferon leads to harmful effects on vascular endothelium in the HSC niche remains to be determined.

MSCs are another key cell type in the HSC niche affected by IFN-γ. MSCs have an important role in HSC maintenance and homeostasis through the production of CXCL12 and stem cell factor (SCF) [62, 72] (Fig. 1a). However, during acute viral infections, IFN-γ produced by cytotoxic T cells stimulates MSC to secrete IL-6 [23•], which is known to skew myeloerythroid differentiation towards myeloid cells [73]. Studies in human BM from AA patients also showed increased expression of IL-6 in stromal cells [44]. Thus, IL-6 signaling, acting downstream of IFN-γ, proves to be yet another cue that pushes HSCs to differentiate (Fig. 1b). Megakaryocytes have been shown to promote quiescence and HSC maintenance through the production of CXCL4 and TGFβ1 [74, 75]. However, megakaryocytes may also promote HSC proliferation during myeloablative stress by secreting fibroblast growth factor 1 (FGF1) [74], opening the possibility of a regulatory role for megakaryocytes in promoting HSC differentiation during immunological stress.

The cellular composition of the niche may also be altered by IFN-γ. McCabe et al. demonstrated that macrophages are negative regulators of HSCs and that IFN-γ signaling promotes HSC suppression by increasing the number of macrophages in the BM [24••] (Fig. 1b). While the mechanisms by which macrophages suppress HSC have not yet been described, a recent paper showed that histamine produced by myeloid-derived cells in the niche promotes HSC quiescence [76••]. These data show that the composition of cell types in the bone marrow niche can exert feedback regulation on HSC behavior. Altogether, the current literature shows that IFN-γ can indirectly affect HSC fate by altering both the function and composition of niche cells.

## IFN-γ in Disease Pathogenesis

While IFN-γ signaling has an important role in HSC embryonic development and homeostasis, it also contributes to the pathogenesis of several diseases. Patients suffering from AA have dysregulated autoreactive T cells, increased blood levels of IFN-γ, and HSC loss likely due to the loss of self-renewal properties [4, 9]. Differentiation of multipotent progenitors (MPP) to granulocyte-macrophage progenitor (GMP) and megakaryocyte-erythroid progenitors (MEP) is also impaired in AA patients by intrinsic IFN-γ inhibition of hematopoiesis [9]. Polymorphisms in intronic regions of IFN-γ transcripts that increase the transcript’s stability and half-life have been correlated with a higher risk of developing AA [77], supporting the observation that prolonged IFN-γ exposure impairs HSC function. Blood levels of IFN-γ are also increased in liver cirrhosis patients, who can develop a cirrhosis-associated immune dysfunction syndrome characterized by impaired hematopoiesis and immunity. It stands to reason that the deleterious effects of persistent inflammation as described above contribute to the hematologic and immuneologic dysfunction seen in these patients [8]. IFN-γ is also a driver of pathogenesis in patients with HLH, a hematological disorder characterized by impaired lymphocyte cytotoxic function and hyperproduction of inflammatory cytokines such as TNF-α, IL-6, and M-CSF, along with IFN-γ [6, 11]. IFN-γ signaling downregulates CD47-SIRPA anti-phagocytic signals, leading to increased hemophagocytosis, anemia, and destruction of HSCs by macrophages [6, 7].

HSC homeostasis is also altered during immune responses against infectious diseases that promote the production of high levels of IFN-γ. Chronic infections such as HIV or tuberculosis, while disparate in nature, both lead to BM suppression [10]. Until recently, the etiology of BM suppression during HIV or tuberculosis have been poorly understood. However, recent studies using mouse models of *M. avium* or viral infection have shown that altered hematopoiesis during these infections can be attributable to the IFN-γ-mediated immune response. For example, chronic *M. avium* infection leads to IFN-γ-dependent irreversible loss of HSC self-renewal and engraftment capabilities, and cytopenias [18••]. Collectively, these studies elucidate the role of IFN-γ in the progression and pathogenesis of diseases that result in HSC suppression.

## Conclusions

In summary, IFN-γ signaling can affect HSC homeostasis directly by altering gene expression in HSCs and indirectly by altering the regulatory roles of neighboring niche cells. Collectively, the studies reviewed here show that short-term IFN-γ signaling induces HSC proliferation, whereas long-term stimulation promotes HSC differentiation and impaired self-renewal. Excessive replicative stress and bias towards terminal differentiation leads to HSC loss during chronic IFN-γ stimulation. Additionally, although IFN-γ alone might be insufficient to trigger HSC apoptosis in the absence of secondary stimulation or stress, IFN-γ sensitizes HSCs to undergo apoptosis [18••, 20, 26••, 44].

Whereas IFN-γ has been implicated in changing the HSC niche and ultimately causing irreversible damage to HSCs [2, 18••, 78], the long-term effects of chronic inflammation in the HSC niche remain unknown. Therefore, whether the loss of HSC self-renewal capabilities is completely or partially due to niche damage remains an important question. The mechanisms by which changes in specific niche cell types (e.g., macrophages) during inflammation alter hematopoiesis remain poorly defined. Answering these questions will expand our understanding of HSC and bone marrow recovery following chronic inflammation.

Finally, the studies reviewed above show that IFN-γ activates HSC differentiation and the subsequent production of myeloid effector cells. Understanding the kinetics of inflammation-induced HSC differentiation could open new opportunities to exploit the therapeutic properties of HSCs and their progeny. Additionally, defining the cues that promote differentiation over self-renewal after IFN-γ stimulation may elucidate potential therapeutic targets in chronic inflammatory diseases that could protect patients against HSC exhaustion and bone marrow failure.
